# Common risk factors for chronic non-communicable diseases among older adults in China, Ghana, Mexico, India, Russia and South Africa: the study on global AGEing and adult health (SAGE) wave 1

**DOI:** 10.1186/s12889-015-1407-0

**Published:** 2015-02-06

**Authors:** Fan Wu, Yanfei Guo, Somnath Chatterji, Yang Zheng, Nirmala Naidoo, Yong Jiang, Richard Biritwum, Alfred Yawson, Nadia Minicuci, Aaron Salinas-Rodriguez, Betty Manrique-Espinoza, Tamara Maximova, Karl Peltzer, Nancy Phaswanamafuya, James J Snodgrass, Elizabeth Thiele, Nawi Ng, Paul Kowal

**Affiliations:** Shanghai Municipal Centre for Disease Control (Shanghai CDC), 1380 Zhongshan Rd (W), Shanghai, 200336 P.R. China; World Health Organization, HIS/HSI/MCS, Geneva, Switzerland; National Center for Chronic and Noncommunicable Disease Control and Prevention (NCNCD), Chinese Center for Disease Control and Prevention (China CDC), Beijing, P.R. China; Department of Community Health, University of Ghana Medical School, Accra, Ghana; National Research Council, Institute of Neuroscience, Padova, Italy; National Institute of Public Health, Mexico City, Mexico; Russian Academy of Medical Sciences, Moscow, Russian Federation; University of Limpopo, Turfloop, South Africa; Mahidol University, Salaya, Thailand; Human Sciences Research Council, Port Elizabeth/Pretoria, South Africa; Office of the Deputy Vice Chancellor: Research and Engagement, Nelson Mandela Metropolitan University, Port Elizabeth, South Africa; Department of Anthropology, University of Oregon, Eugene, Oregon USA; Atlanta, GA USA; Epidemiology and Global Health, Umeå University, Umeå, Sweden; University of Newcastle Research Centre for Gender, Health and Ageing, Newcastle, Australia

**Keywords:** Chronic non-communicable diseases, SAGE, Tobacco use, Obesity, Low- and middle-income countries

## Abstract

**Background:**

Behavioral risk factors such as tobacco use, unhealthy diet, insufficient physical activity and the harmful use of alcohol are known and modifiable contributors to a number of NCDs and health mediators. The purpose of this paper is to describe the distribution of main risk factors for NCDs by socioeconomic status (SES) among adults aged 50 years and older within a country and compare these risk factors across six lower- and upper-middle income countries.

**Methods:**

The study population in this paper draw from SAGE Wave 1 and consisted of adults aged 50-plus from China (N=13,157), Ghana (N=4,305), India (N=6,560), Mexico (N=2,318), the Russian Federation (N=3,938) and South Africa (N=3,836). Seven main common risk factors for NCDs were identified: daily tobacco use, frequent heavy drinking, low level physical activity, insufficient vegetable and fruit intake, high risk waist-hip ratio, obesity and hypertension. Multiple risk factors were also calculated by summing all these risk factors.

**Results:**

The prevalence of daily tobacco use ranged from 7.7% (Ghana) to 46.9% (India), frequent heavy drinker was the highest in China (6.3%) and lowest in India (0.2%), and the highest prevalence of low physical activity was in South Africa (59.7%). The highest prevalence of respondents with high waist-to-hip ratio risk was 84.5% in Mexico, and the prevalence of self-reported hypertension ranging from 33% (India) to 78% (South Africa). Obesity was more common in South Africa, the Russia Federation and Mexico (45.2%, 36% and 28.6%, respectively) compared with China, India and Ghana (15.3%, 9.7% and 6.4%, respectively). China, Ghana and India had a higher prevalence of respondents with multiple risk factors than Mexico, the Russia Federation and South Africa. The occurrence of three and four risk factors was more prevalent in Mexico, the Russia Federation and South Africa.

**Conclusion:**

There were substantial variations across countries and settings, even between upper-middle income countries and lower-middle income countries. The baseline information on the magnitude of the problem of risk factors provided by this study can help countries and health policymakers to set up interventions addressing the global non-communicable disease epidemic.

## Background

Chronic non-communicable diseases (NCDs) are the leading causes of morbidity and mortality in most low- and middle-income countries (LMIC) [1]. Recent estimates demonstrate that nearly 80% of NCDs deaths occur in LMIC and about three fourth of global NCD-related deaths take place after the age of 60 [2]. Behavioral risk factors such as tobacco use, unhealthy diet, insufficient physical activity and the harmful use of alcohol are known and modifiable contributors to a number of NCDs and health mediators [3,4]. Additionally, with over half of the global population in urban areas, risk factors associated with urbanization such as diet, obesity, hypertension, and a decrease in physical activity will all have significant impacts on the health of the population [5]. Self-report activity data document a pattern of increased inactivity with advancing age [6,7]. As part of the English Longitudinal Study on Ageing, Shankar and colleagues found evidence of clustering of health-related behaviors in older adults [8]. Some epidemiological evidence also suggests multiple risk factors were common in rural Africa [9]. Independently or in combination, these risk factors present an opportunity for interventions to reduce future health burdens in ageing populations in LMIC.

The development of a national risk factor profile for NCDs provides key information required for planning prevention and control activities and could also help to predict the future burden of disease. Reliable and comparable analysis of risks to health is especially important for preventing or modifying disease and injury. However, until recently, analysis of health risks were limited by inconsistent methodologies, dated assumptions and/or variations in assessment criteria for evidence on prevalence, causality and hazard size - all of which limited the ability to produce comparable data to estimate population health status [10].

This study used data from the six countries that implemented the World Health Organization’s Study on global AGEing and adult health (SAGE) Wave 1. The purpose of this paper is to describe the distribution of main risk factors for NCDs by socioeconomic status (SES) within and across countries to better understand the levels of modifiable NCD risk factors for adults aged 50 years and older, and whether these risk factors show age, sex, rural/urban, wealth and country-specific differences.

## Methods

### Study design

The study population was drawn from the SAGE Wave 1, which is a longitudinal cohort survey of ageing and older adults from 2007 to 2010 in six low- and middle-income countries (China, Ghana, India, Mexico, Russian Federation and South Africa) [11]. Multistage cluster sampling strategies were used in all countries where, except for Mexico, households were classified into one of two mutually exclusive categories: (1) all persons aged 50 years and older were selected from households classified as ‘50-plus households’; and, (2) one person aged 18–49 years was selected from a household classified as an ‘18–49 household’. The arrangement in Mexico was similar, but included supplementary and replacement samples to account for losses to follow up in selected sampling units since Wave 0 [12]. The sample in India is also representative at the sub-national and sub-state levels for the selected states. Response rates for SAGE countries ranged from 51% in Mexico to 93% in China (India 68%, Ghana 80%, Russia 83%, and South Africa 77%).

### Measures

SAGE used a standardized instrument for collection of sociodemographic information and behavioral risk factors based on the WHO STEPwise approach to Surveillance (WHO STEPS, WHO 2005). This includes alcohol and tobacco consumption, diet and physical activity. In addition, a number of more objective risk factors were assessed, including, waist and hip circumferences, weight, height, and blood pressure.

In our study, alcohol consumption was categorized into two broad groups: non-drinkers and drinkers, with the latter subdivided according to the number of alcoholic drinks consumed during the week before the interview. Heavy drinkers were defined as consuming five or more standard drinks per day for men and four or more standard drinks per day for women.

The Global Physical Activity Questionnaire (GPAQ) was used to measure the intensity, duration, and frequency of physical activity in three domains: occupational; transport-related; and, discretionary or leisure time [13]. The total time spent in physical activity during a typical week, including the number of days and intensity, were used to generate low, moderate, and high categories of physical activity levels.

Tobacco use covered different forms and frequency of tobacco use—manufactured or hand-rolled cigarettes, cigars, cheroots or whether tobacco is smoked, chewed, sucked or inhaled, each day over the week prior to the interview [14].

Information on fruit and vegetable consumption was based on the number of daily servings typically eaten. Sufficient intake was determined according to the number of servings. Five or more servings are considered sufficient, and fewer than five servings are insufficient [15].

Waist and hip circumferences were measured to calculate waist-to-hip ratio [16]. Central obesity can be defined using adult waist-hip ratio (WHR), male WHR more than 0.90 and female WHR more than 0.85.

Blood pressure was measured three times on the right arm/wrist of the seated respondent using a wrist blood pressure monitor. Out of three measurements, an average of the latter two measurements was used as the blood pressure value in this analysis. The definition used to designate hypertension is systolic blood pressure greater than or equal to 140 mmHg and/or diastolic blood pressure greater than or equal to 90 mmHg^19^ and/or self-reported treatment of hypertension with antihypertensive medication currently (the last two weeks before interview) [17].

Weight and height were measured to calculate body mass index (BMI), calculated as weight/height^2^ (kg/m^2^). According to the classification criteria proposed by the WHO [18]. A cut-off point of <18.5 kg/m^2^ is used to define underweight; a BMI of 25–29.9 kg/m^2^ indicates overweight; and a BMI of ≥30 kg/m^2^ indicates obesity. Modified BMI cutoffs for China and India were used to perform an additional set of analyses that describes moderate-to-high risk (BMI 23.0-27.5) and high-to-very high risk (BMI >27.5) in Asian populations [19].

All these seven risk factors were summed, and a new variable representing the cumulative number of risk factors reported/measured for each individual was created, with the range from 0 (no risk factors) to 7 (with all risk factors). SAGE was approved by the World Health Organization's Ethical Review Board as well as a national approval in all six countries. Informed consent has been obtained from all study participants.

### Statistical analysis

SAGE used a stratified multistage-cluster design in each country. Each household and individual was assigned a known non-zero probability of being selected. Household and individual weights were post-stratified according to country-specific population data. Prevalence rates for each risk factor were estimated using post-stratified individual probability weights in each nation to compensate for undercoverage. According to the sampling design of each country, country-specific cluster and/or strata were taken into account to estimate the 95% confidence intervals (CIs). All statistical analyses were conducted using STATA SE version 11 (STATA Corp, College Station, TX).

## Results

A total of 38,670 individuals aged 50 and older participated in the SAGE survey. Individuals who couldn’t completed or partially completed interview or with missing sociodemographic variables were excluded from the analyses. Finally, A total of 34,114 individuals aged 50 and older in the six countries were considered in this analysis. China has the largest sample (N=13,157), and Mexico (N=2,318) the smallest sample. The socio-demographic characteristics for each country are shown in Table 1. The demographic and socioeconomic characteristics of the older population differed widely across the six countries, the proportion of women is higher than men except in the Ghana, which consisted of 52.4% men and 47.6% women. The 50–59 age groups were the highest proportion in all countries. India remained largely a rural society, with more than two-thirds residing in rural areas; in contrast, the majority of older Mexicans, Russians, and South Africans lived in urban areas. Ghana and India had the lowest educational level among the SAGE countries, with over 54% and 51%, respectively, of the older population having no formal education. In contrast, only 0.5% of older Russians had no formal education, and nearly one in five had a college degree or higher.

**Table 1:** **Percent distribution of respondent sociodemographic characteristics, by country and multi-country pooled data, SAGE Wave 1**

	**China**	**Ghana**	**India**	**Mexico**	**Russia**	**South Africa**	**Pooled**
	**(n = 13,157)**	**(n = 4,305)**	**(n = 6,560)**	**(n = 2,318)**	**(n = 3,938)**	**(n = 3,836)**	**(n = 34,114)**
	%	%	%	%	%	%	%
**Age group**							
50-59	44.9	39.7	48.6	48.1	44.1	49.9	45.8
60-69	31.9	27.5	30.9	25.6	26.7	30.6	29.7
70-79	18.6	23.1	16	17.8	21.4	14	18.7
80+	4.6	9.7	4.5	8.6	7.7	5.5	5.8
**Sex**							
Men	49.8	52.4	51	46.8	41.9	44.1	47.2
Women	50.2	47.6	49	53.2	58.1	55.9	52.8
**Residence**							
Urban	47.3	41.1	28.9	78.8	70.1	64.9	50.4
Rural	52.7	58.9	71.1	21.2	29.9	35.1	49.6
**Education level**							
No formal education	23.1	54	51.2	17.2	0.5	25.2	23.8
Less than primary	18.9	10.4	10	38.4	1.2	24	10.1
Primary school completed	21	10.9	14.8	24	5.3	22.4	13.5
Secondary school completed	19.9	4	10.2	9.9	17.9	14.2	16
High school completed	12.6	17.1	8.6	2.4	54.3	8.4	26.2
College completed	4.4	3.4	3.4	5.5	20.7	3.9	9.9
Post graduate degree completed	0.1	0.2	1.7	2.6	0.1	1.8	0.6
Income quintile*							
Lowest	16.3	18.2	18.2	15.3	13.3	20.7	15.9
Second	18.1	19.1	19.5	24.7	17.1	19.9	18.2
Third	20.5	20.5	18.8	16.8	19.6	18.2	19.6
Fourth	23.4	20.7	19.6	16.6	22.1	19.8	21.7
Highest	21.8	21.6	23.9	26.6	27.8	21.3	24.5

*Income levels were generated through a multi-step process, where asset ownership was converted to an asset ladder, a Bayesian post-estimation method used to generate raw continuous income estimates, and then transformed into quintiles. Lowest (Quintile 1) is the quintile with the poorest households and Highest (Quintile 5) the quintile with the richest households.

*Note*: Weighted estimates.

**Figure 1:**
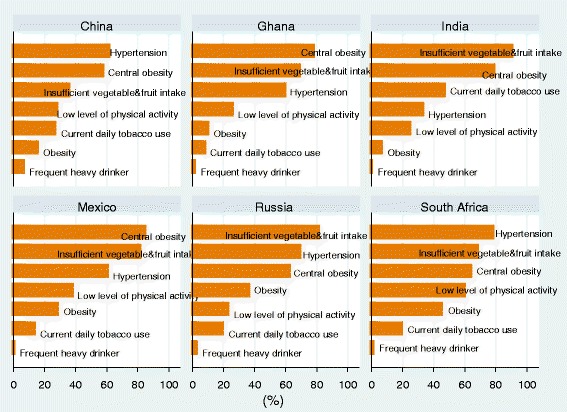
**Ranking of selected risk factors among adults aged 50 years and older across six countries.**

The ranking of all seven NCD risk factors for each country is shown in Figure 1: central obesity, inadequate vegetable fruit intake and hypertension are the three most common risk factors across all six countries, except in India where current daily tobacco use pushed hypertension to fourth among all seven NCD risk factors. In India, the prevalence of inadequate vegetable fruit intake and current daily smoker are the highest among all the six countries. In contrast, the rate of hypertension (33%) in India is the lowest. The prevalence of obesity in Mexico, Russia and South Africa are markedly higher than that in China, India and Ghana.

### Tobacco abuse

The prevalence of daily tobacco use ranged from 7.7% (Ghana) to 46.9% (India). Men were much more likely than women to smoke in all six countries. With increasing age, prevalence of current daily smoker among men decreased in China, and the Russian Federation; however, only minor age differences were seen in Ghana and Mexico. Tobacco use among women declined with age in Mexico and Russia Federation. Older urban residents in China, Ghana, and India were less likely to use tobacco than their rural counterparts, while it was the opposite in Mexico (Table 2).

**Table 2:** **Prevalence of current daily tobacco use by age, sex, rural/urban area and income quintiles among persons aged 50 years and older across six countries**

	**China**		**Ghana**		**India**		**Mexico**		**Russian Federation**		**South Africa**	
	***%***	***95% CI***	***%***	***95% CI***	***%***	***95% CI***	***%***	***95% CI***	***%***	***95% CI***	***%***	***95% CI***
***Men***												
50-59	58.8	[55.3,62.3]	11.1	[9.0,13.6]	63.8	[58.6,68.7]	18.4	[7.2,39.6]	50.7	[39.2,62.1]	25.8	[19.9,32.7]
60-69	50.1	[46.5,53.6]	11.2	[8.8,14.3]	64.3	[59.5,68.8]	19.4	[13.7,26.6]	43.3	[30.4,57.2]	21.4	[21.4,21.4]
70-79	35.1	[31.2,39.3]	10.7	[7.7,14.6]	60.0	[51.3,68.0]	21.1	[12.5,33.5]	14.0	[8.2,23.0]	15.9	[10.6,23.2]
80+	29.8	[23.2,37.3]	13.8	[8.9,20.6]	54.7	[43.6,65.4]	14.6	[7.5,26.5]	5.7	[1.9,15.9]	18.1	[5.7,44.5]
***Women***												
50-59	1.4	[1.0,2.0]	2.0	[1.1,3.5]	26.9	[23.7,30.3]	11.0	[3.6,28.9]	7.9	[5.3,11.6]	17.3	[13.6,21.6]
60-69	3.1	[2.2,4.2]	3.7	[2.4,5.8]	33.5	[28.8,38.6]	8.9	[4.5,17.1]	3.8	[2.1,6.9]	14.9	[11.2,19.6]
70-79	6.1	[4.5,8.2]	6.4	[4.2,9.5]	33.2	[25.5,41.8]	3.6	[2.0,6.7]	2.0	[0.7,5.8]	17.4	[11.5,25.5]
80+	3.7	[1.8,7.5]	3.6	[1.6,7.7]	31.8	[23.3,41.8]	3.3	[1.5,7.1]	0.9	[0.1,5.6]	18.5	[9.7,32.5]
***Residence***												
Urban	19.4	[17.9,21.1]	4.1	[3.0,5.5]	37.1	[31.0,43.6]	15.2	[9.5,23.4]	17.3	[14.4,20.5]	19.2	[16.1,22.9]
Rural	33.4	[31.0,35.9]	10.2	[8.7,11.9]	50.9	[48.4,53.4]	6.3	[3.9,10.2]	24.4	[16.5,34.6]	19.7	[15.7,24.3]
Income quintile*												
Lowest	29.1	[25.6,32.8]	16.0	[12.9,19.7]	57.1	[51.9,62.2]	9.3	[5.6,15.3]	17.9	[11.0,27.6]	20.8	[15.6,27.2]
Second	30.9	[27.5,34.5]	9.1	[7.3,11.4]	54.7	[51.2,58.1]	12.9	[5.8,26.4]	17.1	[11.5,24.7]	17.7	[13.0,23.7]
Middle	26.2	[24.4,28.2]	8.0	[6.0,10.5]	49.8	[45.0,54.7]	11.1	[5.4,21.4]	18.1	[10.9,28.7]	22.3	[17.4,28.1]
Fourth	26.8	[25.1,28.5]	4.8	[3.5,6.5]	43.0	[38.9,47.1]	13.5	[8.2,21.4]	22.3	[14.8,32.1]	18.1	[13.4,24.0]
Highest	21.9	[19.4,24.7]	1.8	[1.0,3.4]	33.5	[29.3,38.1]	17.2	[7.3,35.4]	20.1	[14.4,27.4]	18.2	[13.1,24.7]
***Total***	26.7	[25.3,28.2]	7.7	[6.6,8.8]	46.9	[44.4,49.3]	13.3	[8.6,19.9]	19.4	[16.1,23.3]	19.4	[16.8,22.2]

*Income levels were generated through a multi-step process, where asset ownership was converted to an asset ladder, a Bayesian post-estimation method used to generate raw continuous income estimates, and then transformed into quintiles. Lowest (Quintile 1) is the quintile with the poorest households and Highest (Quintile 5) the quintile with the richest households.

*Note*: Weighted estimates.

### Alcohol consumption

Heavy alcohol consumption was highest in China, where 6.3% of older Chinese were frequent heavy drinkers, compared to just 0.2% of older Indians, the lowest among all six countries. Men were much more likely to drink than women in all countries. For men, the prevalence of heavy alcohol consumption decreased with increasing age in China, Ghana and India. Older rural residents were more likely to drink than their urban dwelling counterparts in all countries, except South Africa (Table 3).

**Table 3:** **Prevalence of frequent heavy drinker by age, sex, rural/urban area and income quintiles among persons aged 50 years and older across six countries**

	**China**		**Ghana**		**India**		**Mexico**		**Russian Federation**		**South Africa**	
	***%***	***95% CI***	***%***	***95% CI***	***%***	***95% CI***	***%***	***95% CI***	***%***	***95% CI***	***%***	***95% CI***
***Men***												
50-59	15.3	[12.7,18.3]	3.2	[2.1,5.0]	0.6	[0.2,1.4]	0		3.9	[1.8,8.5]	1.3	[0.7,2.3]
60-69	12.5	[10.4,15.1]	2.7	[1.6,4.5]	0.4	[0.1,1.0]	0.8	[0.1,5.2]	8.0	[1.7,30.4]	2.1	[1.0,4.5]
70-79	8.5	[6.8,10.6]	1.6	[0.4,5.7]	0		0		0.7	[0.1,4.3]	0	[0.0,0.1]
80+	3.3	[1.6,6.7]	0.4	[0.1,2.8]	0		1.7	[0.3,11.1]	0		0	
***Women***												
50-59	0.5	[0.3,1.1]	0.1	[0.0,0.8]	0.1	[0.0,0.6]	0	-	0	[0.0,0.1]	0.5	[0.2,1.6]
60-69	0.5	[0.2,1.2]	1.2	[0.3,4.5]	0		0	-	2.2	[0.5,9.6]	1.1	[0.2,5.6]
70-79	0.6	[0.3,1.4]	0.2	[0.0,1.7]	0		0	-	0		0.5	[0.2,1.4]
80+	1.6	[0.6,4.6]	0		0		0	-	0		2.6	[0.6,11.4]
***Residence***												
Urban	1.8	[1.3,2.4]	1.2	[0.7,2.1]	0.1	[0.0,0.6]	0.1	[0.0,0.6]	2.2	[0.6,7.3]	1.0	[0.6,1.9]
Rural	10.4	[9.1,11.9]	1.7	[1.1,2.6]	0.3	[0.1,0.7]	0.2	[0.0,1.4]	3.2	[0.8,11.6]	1.0	[0.4,2.3]
Income quintile*												
Lowest	7.0	[5.4,9.1]	1.7	[0.8,3.6]	0.4	[0.1,1.3]	0		2.0	[0.8,4.9]	1.0	[0.4,2.2]
Second	6.9	[5.9,7.9]	1.0	[0.4,2.2]	0.3	[0.1,2.3]	0.3	[0.0,2.0]	8.3	[1.9,30.2]	1.8	[1.8,1.8]
Middle	7.6	[5.7,10.1]	2.2	[1.1,4.3]	0	[0.0,0.2]	0		0.3	[0.1,1.0]	1.1	[1.1,1.1]
Fourth	6.6	[5.2,8.3]	1.6	[0.8,3.2]	0.1	[0.0,0.2]	0.3	[0.0,1.9]	1.2	[0.5,2.8]	0.3	[0.1,1.0]
Highest	4.0	[2.8,5.7]	1.0	[0.5,2.1]	0.2	[0.1,0.7]	0		1.9	[0.5,7.0]	1.0	[0.2,4.4]
***Total***	6.3	[5.6,7.2]	1.5	[1.1,2.1]	0.2	[0.1,0.5]	0.1	[0.0,0.5]	2.5	[1.0,6.1]	1.0	[0.6,1.7]

*Income levels were generated through a multi-step process, where asset ownership was converted to an asset ladder, a Bayesian post-estimation method used to generate raw continuous income estimates, and then transformed into quintiles. Lowest (Quintile 1) is the quintile with the poorest households and Highest (Quintile 5) the quintile with the richest households.

*Note*: Weighted estimates.

### Low level physical activity

Prevalence of low level physical activity was highest in South Africa, at 59.7%. A significant age-gradient was seen in all countries, where prevalence consistently increased with increasing age. Older urban residents were more likely to engage in low level physical activity in all countries (Table 4).

**Table 4:** Prevalence of low level of physical activity* by age, sex, rural/urban area and income quintiles among persons aged 50 years and older across six countries

	**China**		**Ghana**		**India**		**Mexico**		**Russian Federation**		**South Africa**	
	***%***	***95% CI***	***%***	***95% CI***	***%***	***95% CI***	***%***	***95% CI***	***%***	***95% CI***	***%***	***95% CI***
***Men***												
50-59	21.4	[19.1,23.8]	15.9	[12.8,19.5]	14.4	[11.4,18.1]	19.3	[11.2,31.3]	14.6	[9.2,22.3]	49.6	[41.5,57.4]
60-69	26.1	[23.6,28.7]	18.6	[15.0,22.9]	25.0	[21.0,29.6]	32.9	[25.0,41.9]	21.3	[12.7,33.5]	60.7	[53.4,68.8]
70-79	35.8	[31.7,40.0]	29.9	[24.7,35.8]	41.9	[34.1,50.1]	48.0	[36.5,59.8]	33.3	[20.1,49.8]	67.0	[57.2,75.5]
80+	50.2	[43.8,56.6]	37.5	[29.8,46.0]	51.0	[39.9,62.0]	66.8	[55.5,76.5]	50.2	[20.6,79.7]	64.7	[47.0,79.6]
***Women***												
50-59	23.7	[21.4,26.1]	21.3	[17.4,25.8]	17.9	[15.0,21.3]	36.2	[20.7,55.3]	11.1	[6.7,17.8]	56.5	[49.8,62.3]
60-69	28.6	[26.1,31.2]	28.8	[23.5,34.9]	26.8	[22.4,31.6]	46.0	[34.9,57.5]	20.0	[14.4,27.2]	64.9	[57.0,71.2]
70-79	38.4	[34.3,42.7]	39.4	[34.4,44.7]	40.2	[32.7,48.1]	52.9	[37.1,68.1]	32.7	[23.6,43.3]	69.9	[62.3,76.4]
80+	65.1	[58.4,71.3]	43.4	[36.1,51.0]	60.4	[49.4,70.5]	59.3	[42.7,74.1]	66.4	[49.6,79.9]	81.5	[70.9,88.5]
***Residence***												
Urban	28.8	[25.4,32.5]	38.0	[33.5,42.7]	29.8	[24.9,35.2]	39.0	[30.1,48.7]	23.2	[18.8,28.3]	61.2	[55.6,66.4]
Rural	27.8	[26.1,29.7]	17.1	[14.2,20.3]	23.0	[21.1,24.9]	33.0	[22.5,45.5]	22.0	[13.4,33.9]	56.7	[48.9,63.6]
Income quintile**												
Lowest	29.0	[25.8,32.5]	16.9	[14.0,20.1]	23.1	[19.6,27.0]	46.0	[38.7,53.4]	42.4	[29.6,56.3]	60.0	[50.7,68.2]
Second	25.7	[22.9,28.6]	21.0	[17.3,25.3]	24.5	[20.9,28.5]	38.2	[22.0,57.6]	32.5	[25.2,40.8]	59.2	[49.8,67.0]
Middle	26.4	[23.7,29.3]	21.1	[17.8,24.9]	25.3	[20.3,31.2]	27.4	[16.0,42.8]	19.7	[13.6,27.8]	58.0	[50.9,64.3]
Fourth	29.6	[26.7,32.8]	31.2	[26.1,36.7]	27.1	[23.1,31.4]	47.0	[36.3,57.9]	13.5	[9.7,18.4]	63.2	[57.0,69.2]
Highest	30.0	[25.9,34.6]	36.3	[31.1,41.9]	24.7	[21.5,28.1]	33.1	[23.0,45.0]	17.2	[11.1,25.7]	58.1	[50.3,65.9]
***Total***	28.3	[26.4,30.2]	25.6	[23.1,28.3]	24.9	[22.7,27.3]	37.7	[30.3,45.7]	22.8	[18.6,27.7]	59.7	[55.1,63.9]

*High = Vigorous-intensity activity on at least 3 days achieving a minimum of at least 1,500 MET-minutes/week OR 7 or more days of any combination of walking, moderate- or vigorous intensity activities achieving a minimum of at least 3,000 MET-minutes per week;

Moderate = A person not meeting the criteria for the “high” category and: 3 or more days of vigorous-intensity activity of at least 20 minutes per day OR 5 or more days of moderate-intensity activity or walking of at least 30 minutes per day OR 5 or more days of any combination of walking, moderate- or vigorous intensity activities achieving a minimum of at least 600 MET-minutes per week; and,

Low = A person not meeting any of the above mentioned criteria falls in this category.

**Income levels were generated through a multi-step process, where asset ownership was converted to an asset ladder, a Bayesian post-estimation method used to generate raw continuous income estimates, and then transformed into quintiles. Lowest (Quintile 1) is the quintile with the poorest households and Highest (Quintile 5) the quintile with the richest households.

*Note*: Weighted estimates.

### Inadequate fruit and vegetable consumption

Prevalence of inadequate fruit and vegetable intake among India’s older population were relatively higher than any other SAGE country; while China had the lowest prevalence at 35.6%. In China and South Africa, respondents with the highest household income had the lowest prevalence (Table 5).

**Table 5:** Prevalence of insufficient vegetable and fruit intake* by age, sex, rural/urban area and income quintiles among persons aged 50 years and older across six countries

	**China**		**Ghana**		**India**		**Mexico**		**Russian Federation**		**South Africa**	
	***%***	***95% CI***	***%***	***95% CI***	***%***	***95% CI***	***%***	***95% CI***	***%***	***95% CI***	***%***	***95% CI***
***Men***												
50-59	32.0	[27.7,36.7]	67.3	[62.2,72.0]	87.5	[84.4,90.1]	76.4	[48.5,91.8]	80.1	[70.0,87.4]	67.9	[61.8,73.5]
60-69	35.9	[31.0,41.1]	72.3	[67.8,76.4]	86.6	[82.2,90.0]	68.5	[56.9,78.2]	83.2	[70.7,91.1]	60.1	[51.9,67.8]
70-79	41.8	[35.9,47.9]	68.5	[62.7,73.8]	90.9	[87.2,93.7]	79.4	[69.8,86.6]	82.8	[67.4,91.9]	60.3	[49.1,70.6]
80+	49.5	[42.1,56.9]	77.6	[68.7,84.5]	89.5	[82.2,94.0]	86.2	[75.9,92.5]	82.5	[54.4,94.9]	70.1	[53.2,82.9]
***Women***												
50-59	29.4	[25.7,33.4]	65.1	[60.5,69.5]	91.4	[89.1,93.2]	88.5	[80.4,93.5]	77.0	[70.2,82.7]	71.0	[65.9,75.6]
60-69	35.8	[31.3,40.5]	67.4	[62.8,71.7]	95.0	[93.1,96.4]	83.3	[75.9,88.8]	83.7	[75.4,89.5]	73.4	[66.5,79.3]
70-79	41.9	[36.4,47.5]	72.0	[67.3,76.3]	96.4	[93.2,98.1]	84.1	[76.2,89.7]	81.3	[68.8,89.6]	67.2	[58.8,74.7]
80+	64.5	[57.6,71.0]	67.8	[60.4,74.5]	95.3	[91.4,97.5]	90.0	[83.6,94.1]	86.2	[74.6,93.1]	77.9	[65.5,86.8]
***Residence***												
Urban	34.7	[31.0,38.7]	67.1	[63.1,70.9]	88.2	[84.0,91.5]	84.2	[79.2,88.3]	79.7	[70.6,86.6]	65.0	[60.7,69.1]
Rural	36.6	[29.9,43.8]	70.1	[66.5,73.4]	91.6	[90.3,92.8]	70.9	[45.6,87.6]	84.0	[77.4,88.9]	75.1	[68.0,81.1]
Income quintile**												
Lowest	46.6	[37.4,56.1]	75.1	[70.5,79.3]	95.7	[94.0,96.9]	89.1	[83.5,93.0]	84.3	[72.3,91.7]	75.3	[66.7,82.2]
Second	42.0	[34.8,49.5]	70.4	[66.2,74.3]	95.3	[93.2,96.8]	79.8	[52.4,93.5]	72.6	[57.4,83.9]	73.7	[66.2,79.9]
Middle	36.7	[32.1,41.5]	68.8	[64.4,72.9]	92.4	[89.2,94.8]	82.2	[69.9,90.2]	78.1	[67.6,85.9]	69.5	[63.5,74.9]
Fourth	30.2	[26.3,34.5]	67.5	[62.5,72.1]	88.1	[85.4,90.4]	76.7	[69.1,82.9]	82.4	[74.9,88.0]	69.6	[62.9,75.5]
Highest	26.8	[22.7,31.4]	63.7	[59.2,67.9]	83.5	[79.6,86.8]	80.7	[68.9,88.8]	85.5	[78.3,90.7]	54.9	[47.9,61.7]
***Total***	35.6	[31.6,39.8]	68.9	[66.2,71.4]	90.6	[89.1,91.9]	81.4	[74.1,87.0]	81.0	[74.5,86.2]	68.4	[64.6,72.0]

*Insufficient intake is equivalent to less than 5 servings of fruit and vegetables on average per day.

**Income levels were generated through a multi-step process, where asset ownership was converted to an asset ladder, a Bayesian post-estimation method used to generate raw continuous income estimates, and then transformed into quintiles. Lowest (Quintile 1) is the quintile with the poorest households and Highest (Quintile 5) the quintile with the richest households.

*Note*: Weighted estimates.

### Central obesity

Central obesity was found in 84.5% of older Mexicans, the highest of all SAGE countries. In China and Ghana, prevalence tended to increase with age, and was higher in urban than in rural areas. The most eye-catching difference is the much higher implied risk among women compared to men in China, Ghana, India and South Africa. Patterns by level of household income were mixed (Table 6).

**Table 6:** Prevalence of central obesity * by age, sex, rural/urban area and income quintiles among persons aged 50 years and older across six countries

	**China**		**Ghana**		**India**		**Mexico**		**Russian Federation**		**South Africa**	
	***%***	***95% CI***	***%***	***95% CI***	***%***	***95% CI***	***%***	***95% CI***	***%***	***95% CI***	***%***	***95% CI***
***Men***												
50-59	41.4	[38.6,44.3]	61.8	[57.4,66.0]	74.1	[70.6,77.2]	96.3	[92.3,98.3]	69	[53.5,81.2]	54.2	[48.5,59.7]
60-69	48.3	[45.0,51.6]	68	[63.7,72.0]	76.2	[72.3,79.8]	85.9	[68.7,94.4]	69.2	[47.0,85.1]	61.4	[53.7,68.6]
70-79	51.2	[46.8,55.7]	72.2	[66.9,77.0]	66.4	[58.2,73.7]	90.8	[84.4,94.8]	74	[55.9,86.5]	53.5	[42.9,63.8]
80+	56.2	[48.3,63.9]	74	[65.8,80.8]	83.2	[73.7,89.8]	84.6	[74.3,91.3]	42.3	[15.5,74.6]	49.7	[31.7,67.7]
***Women***												
50-59	63.7	[60.6,66.7]	88.9	[86.3,91.1]	81.4	[78.1,84.2]	84.3	[72.5,91.6]	48.8	[40.5,57.2]	67.8	[62.4,72.7]
60-69	70.9	[67.5,74.1]	89.3	[86.3,91.7]	86.7	[83.5,89.3]	81.4	[74.5,86.8]	65.7	[56.9,73.6]	70.8	[63.7,76.9]
70-79	74.9	[70.7,78.7]	90.2	[86.6,93.0]	86.3	[81.3,90.1]	61.1	[40.9,78.1]	61.4	[47.1,73.9]	76.1	[69.0,82.0]
80+	75.4	[68.9,80.9]	90.6	[85.9,93.8]	84.6	[75.8,90.5]	72.9	[56.8,84.6]	75.2	[61.6,85.1]	74.9	[61.8,84.6]
***Residence***												
Urban	61.4	[58.1,64.6]	78.2	[75.3,80.8]	82.9	[78.9,86.2]	84.3	[78.6,88.7]	62.9	[56.4,68.9]	64.7	[60.7,68.4]
Rural	54.1	[51.3,57.0]	77.2	[75.2,79.2]	77.1	[75.2,79.0]	85.2	[72.3,92.7]	60.2	[46.7,72.3]	62.9	[57.8,67.7]
Income quintile**												
Lowest	59.5	[56.3,62.6]	75.6	[72.3,78.7]	74.8	[70.4,78.7]	84.2	[75.3,90.4]	65.2	[53.9,75.0]	59.4	[52.3,66.1]
Second	53.6	[49.8,57.3]	78.5	[74.7,81.8]	75.7	[71.7,79.2]	81.1	[67.5,89.9]	61.2	[51.7,69.9]	59.8	[53.5,65.8]
Middle	56.1	[52.4,59.7]	77.8	[73.9,81.2]	76.6	[73.0,79.9]	88.8	[80.6,93.8]	56.6	[46.4,66.2]	67.8	[61.6,73.5]
Fourth	56.7	[54.0,59.2]	77.4	[73.2,81.1]	79.5	[75.6,82.9]	87	[80.7,91.5]	66	[57.3,73.6]	67.5	[60.6,73.7]
Highest	61.4	[57.2,65.5]	78.6	[75.3,81.5]	85.2	[82.1,87.9]	83.7	[71.9,91.1]	61.9	[49.3,73.1]	65.6	[58.0,72.4]
***Total***	57.4	[55.2,59.6]	77.6	[75.9,79.2]	78.7	[77.0,80.4]	84.5	[79.5,88.5]	62.1	[56.1,67.7]	63.9	[60.7,67.0]

*High-risk waist to hip ratio: men more than 0.90 and women more than 0.85.

**Income levels were generated through a multi-step process, where asset ownership was converted to an asset ladder, a Bayesian post-estimation method used to generate raw continuous income estimates, and then transformed into quintiles. Lowest (Quintile 1) is the quintile with the poorest households and Highest (Quintile 5) the quintile with the richest households.

*Note*: Weighted estimates.

### Hypertension

Prevalence of hypertension in six countries ranged from 33% (India) to 78% (South Africa). For both men and women in China, India and Russia, prevalence of hypertension increased with age. Prevalence were higher in urban than in rural areas in Ghana, India and Mexico. In China, prevalence decreased with increasing household income. But in Ghana and India, respondents with higher household income were more likely to have higher prevalence of self-report hypertension (Table 7).

**Table 7:** Prevalence of hypertension* by age, sex, rural/urban area and income quintiles among persons aged 50 years and older across six countries

	**China**		**Ghana**		**India**		**Mexico**		**Russian Federation**		**South Africa**	
	***%***	***95% CI***	***%***	***95% CI***	***%***	***95% CI***	***%***	***95% CI***	***%***	***95% CI***	***%***	***95% CI***
***Men***												
50-59	53.8	[51.0,56.5]	56.9	[52.3,61.3]	29.3	[26.2,32.6]	49.1	[32.5,66.0]	61.5	[49.6,72.1]	70.5	[65.1,75.5]
60-69	64	[60.6,67.3]	58.2	[52.8,63.4]	29.7	[25.2,34.6]	67.1	[57.7,75.2]	68.9	[59.9,76.7]	79.6	[73.2,84.8]
70-79	69.5	[65.5,73.2]	59.2	[53.3,64.8]	35.9	[28.3,44.3]	70.7	[59.9,79.6]	72	[55.9,83.9]	80	[70.6,87.0]
80+	78.1	[72.4,82.9]	51.7	[43.9,59.5]	40.1	[30.0,51.3]	70.6	[58.2,80.6]	87.9	[74.4,94.8]	74	[57.5,85.7]
***Women***												
50-59	53	[49.6,56.3]	61	[56.6,65.2]	30.8	[26.9,35.1]	48	[29.6,66.9]	57	[49.2,64.5]	78.9	[74.3,82.9]
60-69	65.8	[62.0,69.3]	62.1	[57.2,66.7]	37.2	[32.5,42.1]	65.3	[52.0,76.6]	77.7	[69.6,84.2]	81.3	[75.3,86.1]
70-79	72.4	[68.7,75.8]	61.9	[56.5,67.0]	43.2	[37.2,49.4]	84.8	[77.7,89.9]	82.1	[71.5,89.4]	84.5	[78.2,89.2]
80+	74	[66.5,80.4]	60.5	[53.1,67.4]	40.3	[30.3,51.2]	75.9	[62.1,85.8]	88.9	[80.7,93.8]	83.9	[75.2,89.9]
***Residence***												
Urban	58.8	[56.3,61.3]	67.1	[63.8,70.2]	36.8	[30.8,43.4]	59.5	[51.8,66.8]	70.1	[64.7,75.0]	77.7	[74.6,80.5]
Rural	63.6	[60.2,66.9]	53.7	[50.4,57.0]	31.5	[29.8,33.3]	63.5	[46.8,77.5]	66.9	[60.7,72.5]	78.8	[74.5,82.6]
Income quintile**												
Lowest	64.6	[60.4,68.7]	50.7	[45.7,55.6]	27.4	[23.3,31.9]	65.2	[58.1,71.7]	72.4	[61.7,81.1]	75.6	[69.4,80.9]
Second	60.3	[56.6,63.9]	56.7	[52.3,60.9]	30.9	[27.3,34.8]	70.6	[51.4,84.5]	74.8	[67.5,80.9]	77.4	[71.2,82.6]
Middle	60.7	[58.3,63.1]	58	[54.0,62.0]	30.3	[26.2,34.7]	48.5	[27.8,69.6]	71.6	[61.3,80.0]	80.2	[75.2,84.5]
Fourth	62.1	[59.4,64.8]	62.6	[58.5,66.6]	34.2	[30.5,38.1]	54	[42.3,65.3]	70.6	[62.4,77.7]	77.8	[71.9,82.8]
Highest	59.3	[55.3,63.2]	66.5	[62.4,70.3]	40.2	[36.3,44.2]	59.3	[44.9,72.3]	60.8	[50.2,70.5]	79.2	[74.9,83.0]
***Total***	61.3	[59.0,63.6]	59.2	[56.8,61.5]	33	[31.0,35.1]	60.3	[53.4,66.9]	69.2	[64.9,73.2]	78	[75.6,80.3]

*Hypertension defined as systolic blood pressure greater than or equal to 140 mmHg and/or diastolic blood pressure greater than or equal to 90 mmHg19 and/or self-reported current treatment (in previous two weeks) of hypertension with antihypertensive treatments.

**Income levels were generated through a multi-step process, where asset ownership was converted to an asset ladder, a Bayesian post-estimation method used to generate raw continuous income estimates, and then transformed into quintiles. Lowest (Quintile 1) is the quintile with the poorest households and Highest (Quintile 5) the quintile with the richest households.

*Note*: Weighted estimates.

### Obesity

Obesity was more common in South Africa, the Russia Federation and Mexico (45.2%, 36%, and 28.6%, respectively) compared with China, Ghana and India (15.3%, 9.7%, and 6.4%, respectively). Obesity tended to rise with household income in all six countries, but a slight drop can be seen for the highest income quintile in China, Mexico, Russia Federation and South Africa (Table 8).

**Table 8:** Prevalence of obesity* by age, sex, rural/urban area and income quintiles among persons aged 50 years and older across six countries

	**China**		**Ghana**		**India**		**Mexico**		**Russian Federation**		**South Africa**	
	***%***	***95% CI***	***%***	***95% CI***	***%***	***95% CI***	***%***	***95% CI***	***%***	***95% CI***	***%***	***95% CI***
***Man***												
50-59	11.8	[10.1,13.7]	7.1	[5.4,9.3]	5.3	[3.5,8.0]	22.8	[12.8,37.4]	34.4	[21.2,50.6]	36.5	[30.8,42.5]
60-69	12.6	[10.6,14.9]	6.5	[4.6,9.1]	2.8	[1.6,5.1]	23.4	[16.8,31.4]	18.5	[9.6,32.8]	43.2	[35.0,51.7]
70-79	10.6	[8.3,13.5]	4.8	[2.8,8.3]	3.3	[1.9,5.7]	17.3	[11.2,25.7]	33.2	[20.1,49.7]	37.4	[27.3,48.7]
80+	9.9	[6.4,15.1]	5.5	[2.3,12.7]	4.0	[1.1,13.8]	16.7	[7.9,31.8]	7.7	[2.6,20.7]	30.7	[18.3,46.7]
***Woman***												
50-59	19.7	[17.8,21.6]	19.5	[15.6,24.1]	10.3	[8.4,12.4]	40.4	[23.9,59.4]	46.6	[40.0,53.4]	53.2	[48.2,58.3]
60-69	19.5	[17.1,22.2]	12.3	[9.8,15.5]	8.1	[5.9,10.9]	36	[27.2,45.8]	44.0	[34.7,53.8]	55.2	[49.0,61.3]
70-79	18.2	[15.0,21.9]	8.2	[5.8,11.4]	6.0	[3.2,11.2]	23.9	[15.3,35.3]	34.1	[24.82,44.9]	40.0	[31.4,49.4]
80+	10.5	[6.8,15.8]	6.4	[3.3,12.1]	3.5	[1.7,7.0]	19.6	[12.0,30.3]	28.9	[18.3,42.5]	33.5	[23.0,46.0]
***Residence***												
urban	17.4	[15.7,19.3]	17.6	[14.8,20.9]	12.1	[9.3,15.6]	30.5	[23.3,38.9]	35.9	[30.3,42.0]	47.2	[42.8,51.7]
rural	13.7	[11.8,15.9]	4.3	[3.4,5.4]	4.1	[3.4,4.9]	21.8	[15.8,29.3]	36	[25.9,47.5]	41.2	[35.1,47.6]
***Income quintile*** ^**^												
Lowest	9.0	[6.8,11.9]	2.7	[1.6,4.3]	1.4	[0.8,2.4]	21.0	[14.6,29.1]	31.7	[23.9,40.8]	36.1	[28.1,44.9]
Second	12.8	[11.0,14.8]	4.0	[2.7,6.0]	4.9	[1.4,15.9]	27.9	[14.8,46.1]	31.8	[22.8,42.3]	40.5	[34.6,46.7]
Middle	16.3	[14.9,17.9]	7.0	[5.3,9.3]	4.0	[2.6,6.0]	28.8	[15.9,46.4]	29.7	[22.4,38.3]	48.6	[42.3,55.0]
Fourth	18.1	[16.5,19.8]	10.7	[8.6,13.3]	4.6	[3.4,6.4]	34.3	[24.4,45.7]	43.3	[32.0,55.3]	55.6	[49.3,61.8]
Highest	18.4	[16.4,20.6]	22.3	[18.4,26.9]	14.5	[11.7,17.9]	30.1	[19.8,42.9]	38.8	[29.1,49.6]	46.2	[38.9,53.7]
***Total***	15.3	[13.9,16.8]	9.7	[8.4,11.2]	6.4	[5.2,7.7]	28.6	[22.8,35.3]	36.0	[30.9,41.3]	45.2	[41.6,48.9]

* BMI ≥30 kg/m^2^ or BMI >27.5 kg/m^2^ in China and India.

** Income levels were generated through a multi-step process, where asset ownership was converted to an asset ladder, a Bayesian post-estimation method used to generate raw continuous income estimates, and then transformed into quintiles. Lowest (Quintile 1) is the quintile with the poorest households and Highest (Quintile 5) the quintile with the richest households.

*Note*: Weighted estimates.

### Multiple risk factors

**Figure 2:**
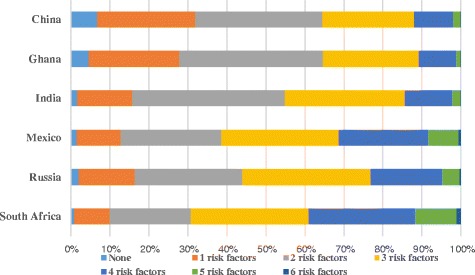
**Percentage of cumulative risk factors among persons aged 50 years and older across six countries.**

Different combinations of risk factors were found. China, Ghana and India had a higher prevalence of respondents with one risk factor than Mexico, Russia Federation and South Africa. Analysis of combinations of two risk factors indicated a less marked difference between the two groups of countries. The occurrence of three and four risk factors was more prevalent in Mexico, Russia Federation and South Africa (see Figure 2).

## Discussion

This is, to our knowledge, the first population-based comparative paper of NCD risk factors specifically designed for older adults residing in LMIC. Participating SAGE countries, China, India, the Russian Federation and South Africa are part of the BRICS countries. Being the biggest countries in the world, China and India together constitute about 38% of the world’s population aged 50 years and older [20]. According to the World Bank, GDP per capita was highest in the Russian Federation, Mexico and South Africa, followed by China, India and Ghana was lowest among all the six countries in 2010. This study reports the prevalence of seven common risk factors for NCDs and demonstrated differences in prevalence across six countries as well as variations within countries. The data were collected using a standard protocol to ensure the comparability of data, the same equipment were used to measure weight, height, waist and hip circumferences, and blood pressure to minimize a possible bias in the measurements.

We found that central obesity, inadequate vegetable fruit intake and hypertension are the most common risk factors for NCDs across all six countries except India, where current daily tobacco use replaced hypertension. The highest burden of hypertension was found in South Africa and the Russian Federation, with 78% and 69%, respectively, followed by China, Ghana and Mexico, all over 50%. These figures seem to be higher than previously found among older adult populations in Africa (rural Malawi, Rwanda and Tanzania (36.6–41.0%) [9], 42.4% of women in Accra, Ghana [21] and East Asia, China (24.2–64.9%) [22,23], and Taiwan (31.1–38.0%) [24]. In addition, the prevalence and awareness of hypertension in urban and rural dwellers in SAGE Wave 1 show marked differences, especially those on treatment and with adequate control by age and urban or rural residence. Individuals not diagnosed but with high blood pressure on measurement (higher in rural settings) are as much of a concern as those who know they have hypertension and are still hypertensive on measurement (much higher in urban settings). In these six countries, only 4–14% were receiving effective treatment [25].

The results of this study also show that older adults from upper-middle income countries such as Mexico, Russian Federation and South Africa are more likely than those from low or lower-middle income countries such like China, India and Ghana to be obese. South Africa has the highest prevalence of obesity (45.2%), even higher than Europeans aged 50 years and older [26], especially among those aged 60–69 years (50%) and among urban dwellers (47%). Over nutrition play an important role and determinants include female gender, low physical activity and chronic conditions [27]. Obesity seems less of a concern for old adults in China and India for now compared to other four countries, although obesity has increased 4-fold in the last 2 decades in china [28]. Like the pattern of prevalence of obesity, the prevalence of low physical activity was also highest in South Africa and Mexico. As the association between physical inactivity and obesity is well recognized [29,30], low physical activity was a very important factor contributing to obesity in these two countries, but was not found in the Russian Federation in this study, where it has lower prevalence of low physical activity compared to Mexico but has higher prevalence of obesity, thus indicating that other health behavior such as alcohol consumption and/or socioeconomic factors related to nationality are influencing obesity [31].

Tobacco use is serious health-damaging behavior in China [32,33], this study seems to have again confirmed. We also found prevalence of current daily tobacco use among older Chinese men was close to the GATS with self-reported prevalence of 58.9% and 40.2% among adults aged 55–64 years and 65 years plus, respectively [34]. Tobacco use is also very prevalent in India, with almost half of Indian are current daily smoker in this study, It is worth noting that smokeless tobacco use is particularly prevalent in India, which is different from other five countries. However, there is evidence that smokeless tobacco use plays a role in oral cancer in south-central Asia [35]. About 52% of oral cancers in India are attributable to the use of smokeless tobacco products [36]. No evidence show rates of smoking are decreasing in LMIC. Suggesting health policy, planning and programmes of tobacco control should promote implementation of effective strategies [37].

Analysis of the simultaneous occurrence of more than one risk factor indicates that people aged 50 years and older across six countries engage in a number of risk factors that put them at high risk of NCDs, however, we found that these selected risk factors occurred much more frequently in upper-middle income countries than in low-middle income countries. This difference may reflect the fact that compared with older adults in upper middle-income countries, older adults in lower middle-income countries are more likely to have had lower levels of exposure to NCD-risk factors associated with urban living (such as smoking, sedentary lifestyles and processed foods) [38].

We also found the pattern of associations between income and risk factors for NCDs vary among countries. The association of income with smoking has been reported before in other studies on Western societies [39-41]. We found that the pattern of tobacco use association with household wealth differed between low-middle income countries and upper-middle income countries in this study. Wealth showed a strong relationship with current daily smoking in low-middle income countries such as Ghana and India, but it does not show any specific trend with income in upper-middle income countries such as Mexico, the Russian Federation and South Africa. Previous studies have shown that education is more strongly related to smoking than income is in most countries within the European Union [42], considering education and economic status are closely related in developing countries, so this may in part explain this difference between upper-middle income countries and low-middle income countries. There is a lot of controversy on association between income and obesity, numerous studies show that low-income and obesity are linked in many high income nations [43]. But results of this study show inverse pattern of association between income and obesity, that increasing income increased the risk of obesity. There is also still little difference between upper-middle income countries and low-middle income countries. The prevalence of obesity reach their peak among older adults in the fourth income quintile in South Africa, Mexico, Russia Federal and China, but occurred in the highest income quintile in Ghana and India. This implies that the burden of obesity is shifting toward the low SES and can no longer be considered a disease of the socioeconomic elite in LMIC [44]. We observe what appears to be the first inkling of the transition in South Africa, Mexico, the Russian Federation and China. It implies policymakers in developing countries and even low-income countries should prepare in advance to address this transition over the next several decades [45-48]

Some limitation must be taken into account in this study. First, there are different response rate across six countries,from 51% in Mexico to 93% in China. The low response rate was potential selection bias to this study. The main reason for household non-response was inability to locate the selected household, or the household refusing to participate even before a roster could be obtained. Second, a limitation to this study is the use of self-report for part of risk factors for NCDs. It can lead to recall bias, although self-reported method widely applied in population study and other studies have illustrated the reliability and validity of self-report for behaviors such as cigarette smoking, alcohol consumption, and physical activity [49,50]. Finally, SAGE wave 1 is a cross-sectional study, which determines that we could not examined the changes in prevalence of these risk factors for NCDs over time, fortunately, SAGE Second and third waves of data collection will be 2013 and 2015. It will provide an opportunity to track these changes.

## Conclusions

In conclusion, this study estimated the prevalence rates of common risk factors for NCDs and showed the pattern of these risk factors in six main LMIC. The baseline information on the magnitude of the problem of risk factors provided by this study can help countries and health policymakers to set up interventions addressing the global non communicable disease epidemic. Understanding the relationship of risk factors pattern and burden of NCDs in LMIC presents an important challenge for further research.

## Abbreviations

NCDs: Chronic non-communicable diseases
SES: socioeconomic status
SAGE: Study on global AGEing and adult health
LMIC: low- and middle-income countries
GPAQ: The Global Physical Activity Questionnaire
WHR: waist-hip ratio
